# Environmental and geographical conditions influence color, physical properties, and physiochemical composition of pomegranate fruits

**DOI:** 10.1038/s41598-023-42749-z

**Published:** 2023-09-18

**Authors:** Ali Akbar Ghasemi-Soloklui, Mojtaba Kordrostami, Ali Gharaghani

**Affiliations:** 1 Nuclear Agriculture Research School, Nuclear Science and Technology Research Institute (NSTRI), Karaj, Iran; 2 Department of Horticultural Science, School of Agriculture, Shiraz University, Shiraz, Iran

**Keywords:** Agroecology, Plant physiology, Fruiting

## Abstract

The highest quality pomegranate necessitates a tropical or subtropical environment for proper growth and development. This study evaluated two pomegranate cultivars including Rabab Poost Ghermez Neyriz (RPGN) and Makhmal Malas Shahreza (MMS) for physical traits, biochemical properties, and juice quality in their native locations as well as other warm and arid regions during two growing seasons (2019–2020 and 2020–2021) in Iran. The results showed that cultivars with the maximum redness (a*) were more likely to originate in cooler climates, and the cultivar’s responses to changing climates were also different. According to pomegranate characteristics, cultivars in different regions had different fruit, aril, and skin weights. According to these findings, pomegranate fruits cultivated in other climates than the origin climate have a smaller edible fraction. The findings also demonstrate that pomegranate fruits cultivated in mountain climates have more significant biochemical parameters such as total phenol, anthocyanin content, antioxidant capacity, and vitamin C than those produced in desert environment settings. The increased titratable acidity (TA), total soluble solids (TSS), and pH values of pomegranates produced in origin climate than the warm environment; thus, suggest that changes in pomegranate cultivar origin had a clear impact on fruit juice quality. Environmental factors, such as wind speed, altitude, and annual precipitation, had a significant correlation with a* skin, TSS, fruit weight, aril weight, edible portion, pH, TA, phenol, antioxidants, and anthocyanin content.

## Introduction

In addition to its long history of domestication in Iran and other parts of Central Asia, pomegranates (*Punica granatum* L.) have spread to other warm locations throughout the globe due to trade and invasion^1^. It’s one of the most economically viable tropical and subtropical fruit crops, especially in arid and semi-arid regions with poor soil and water quality and other yield-restricting variables^2^. Temperature and humidity in the summer are critical for the pomegranate to mature correctly; in addition, the tree’s distribution is determined by its vulnerability to cold temperatures.

As far as pomegranate production goes, it is primarily found in southern and central Asia (Iran, India, China, Syria, Afghanistan, Turkmenistan, and Pakistan), the Mediterranean basin (Spain, Italy, and Morocco), and the desert regions of California and Arizona (California and Arizona). Due to its high economic value and increased demand in international markets for pomegranate products, mainly minimally processed fresh arils and juice, as the health benefits of pomegranates have been revealed, worldwide cultivation and production of pomegranates have increased significantly in recent years. In regions where pomegranates are grown swiftly, environmental conditions that cannot be avoided may impact the quality and quantity of the fruit. The pomegranate is a tropical and subtropical fruit tree that thrives in the right conditions to provide the most delicate fruit. However, the highest fruit quality and quantity are grown in climatic conditions with moderate winters, dry, hot summers without rainfall in late summer/autumn, and sunny days, of course^3^. Pomegranate fruit may be damaged by sunburn when the temperature becomes too hot during the summer. According to prior research, changes in rainfall, temperature, and solar radiation, among other crucial climatic and meteorological variables, significantly impact apple development and crop yields^4–6^. In addition, Abobatta^7^ found that climatic parameters such as temperature, heat stress, CO_2_ assimilation, rainfall, and humidity may influence fruit citrus output directly and indirectly. Climate change affects the texture, taste, and color of the fruit and other aspects of fruit quality. Sthapit et al.^8^ found correlations between the evolutionary origin of a particular cultivar and the physiological responses of tropical fruit trees to environmental conditions. For the optimum pomegranate taste and color, high temperatures throughout the fruiting development stage were cited by Glozer and Ferguson^9^ Pomegranate cultivars from Spain were equally vulnerable to environmental influences on fruit color intensity, according to Holland et al.^10^. Pomegranate fruit and aril have been shown to vary in biochemical composition and mineral components based on genetics, growing location, climatic conditions, maturation, harvest procedures, and storage conditions. In addition, commercial Iranian pomegranate cultivars vary significantly in color, size, and several biochemicals including TSS, TA, pH, phenol content, and antioxidants^2^. Consequently, the concentration of these chemicals in pomegranate fruit is critical to meet the high demand for various goods by the marketing and processing industries. Even with new pomegranate orchards springing up throughout the globe and rising worldwide demand for high-quality pomegranates, Iran remains the world's leading producer and supplier of fruit. As a result, better knowledge of environmental influences on fruit quality and quantity is essential, especially in the case of Iran, which is particularly sensitive to the harmful effects of climate change and global warming.

Aims of the research included evaluating the physical characteristics (skin and aril color, fruit weight, aril weight, and edible portion), biochemical characteristics (antioxidant capacity, TSS, phenol content, and vitamin C and anthocyanin content), as well as the correlation analysis between fruit quality traits, climatic conditions and geographical factors in two cultivars of pomegranate during two growing seasons (2019–2020 and 2020–2021), including RPGN and MMS in them origin area and other regions (desert climate) of Iran.

## Results

### Fruit skin and aril color

**Figure 1:**
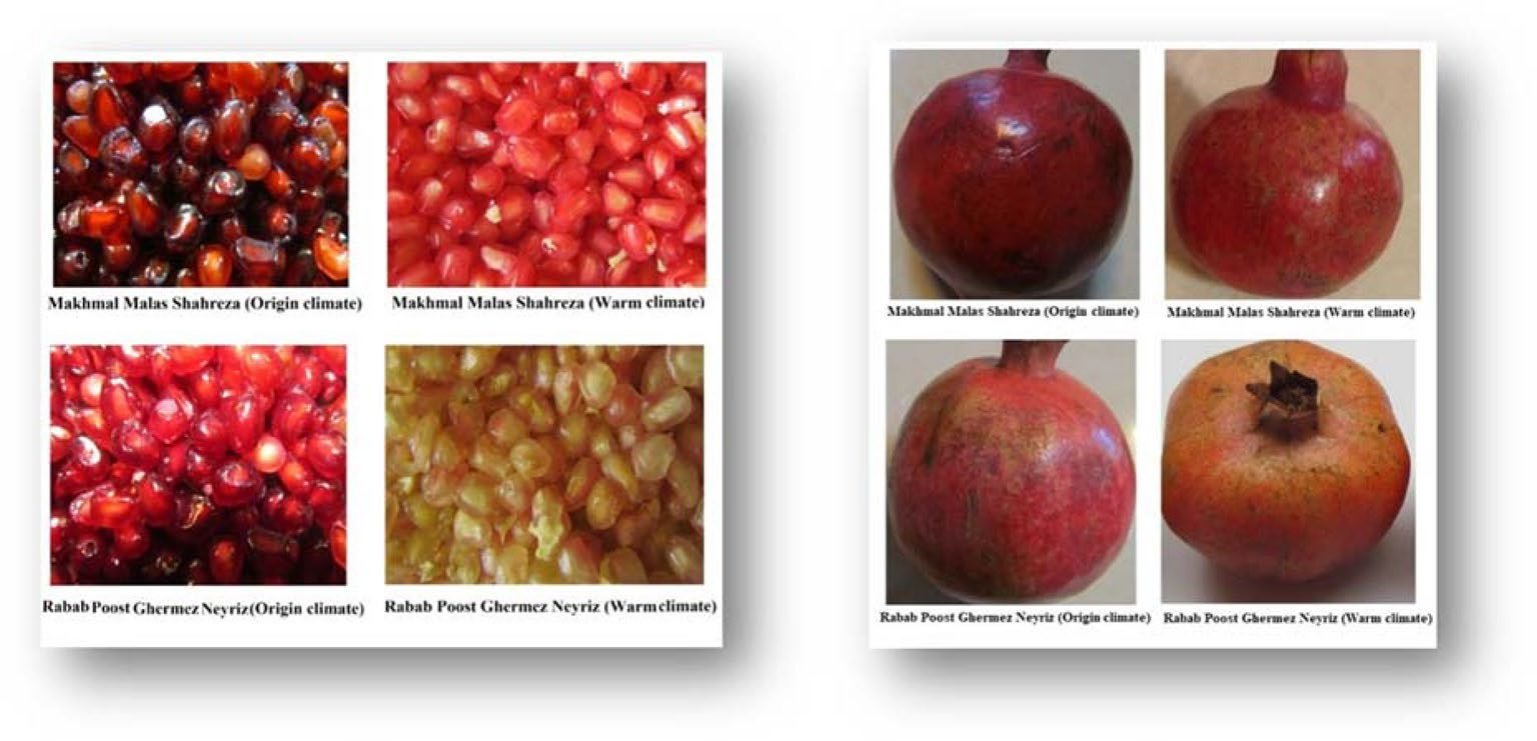
Effects of cultivars origin change on aril and skin color of two pomegranate cultivars.

Table 1 summarizes the color attributes of skin pomegranate, the skin a* values varied from 8.33 to 18.08, with the largest a* values recorded at the origin site of both cultivars. On the other hand, the* value of skin color was reduced in warm temperature cultivars, particularly MMS, while the lightness (L*) and yellowness (b*) of the skin color index rose from the origin site to the warm-climate climate. RPGN cultivar also had the most unique L* and b* skin color in warm conditions (Table 1). According to this research, the maximum redness (a*) cultivars were associated with the origin climate rather than a warm environment, with the most reduced a* value (40.8 to 22.60) recorded in RPGN. Changes in the origin of pomegranate cultivars influence the L* and b* values of the aril color index, similar to skin color (Table 1). In the warm climate was observed to have the highest L* (53.16 and 42.16) and b* (23.44 and 16.2) in two cultivars, particularly MMS. Changing the origin of pomegranate cultivars affects the color of the fruit skin and the color of the aril in pomegranate cultivars (Fig. 1).

**Table 1:** Effects of cultivars origin change on skin and aril color in pomegranate (mean of the 2-year data).

Cultivars	L*		a*		b*	
	Origin climate	Warm climate	Origin climate	Warm climate	Origin climate	Warm climate
Aril color						
Rabab Poost Ghermez Neyriz	8.81 ± 1.2	36.58 ± 4	10.83 ± 0.76	8.33 ± 1.04	4.49 ± 0.18	11.06 ± 0.75
Makhmal Malas Shahreza	10.77 ± 1.59	22.5 ± 0.9	18.08 ± 1.38	14.77 ± 2.33	2.51 ± 0.22	9.24 ± 0.29
Skin color						
Rabab Poost Ghermez Neyriz	40.92 ± 1.45	53.16 ± 1.54	40.85 ± 1.24	22.60 ± 3.26	15.16 ± 0.64	23.44 ± 0.88
Makhmal Malas Shahreza	36.74 ± 0.82	42.16 ± 0.73	39.70 ± 0.32	34.39 ± 1.96	11.78 ± 0.56	16.2 ± 0.84

Similar letters at the top of the rows indicate non-significant differences at P ≤ 0.05.

### Fruit physical properties

**Figure 2:**
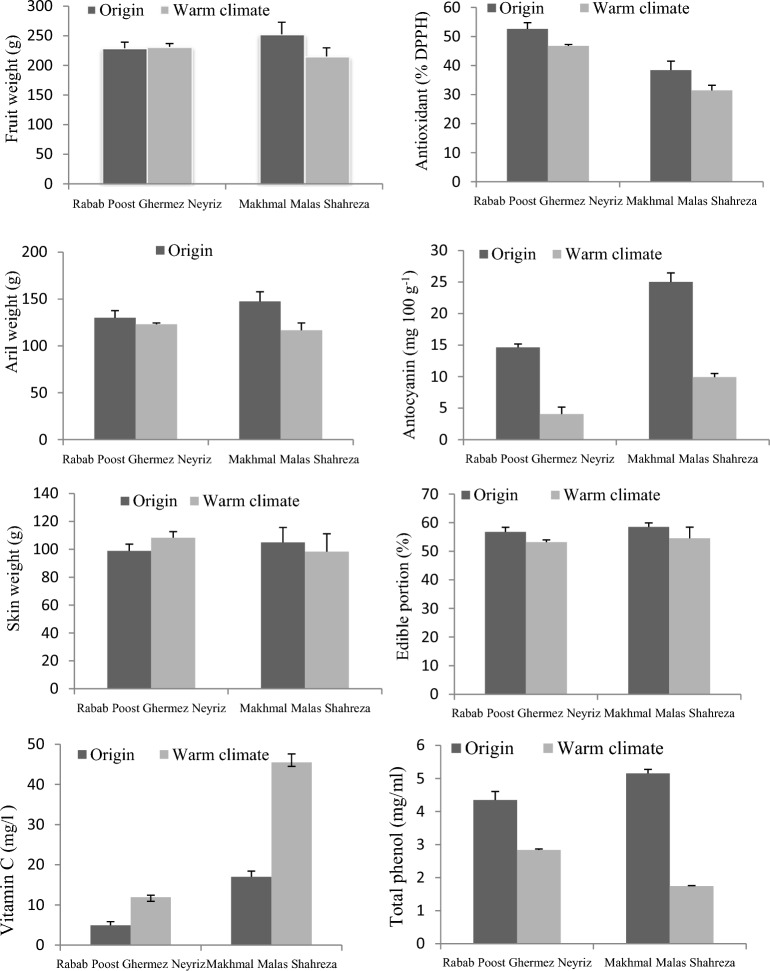
Effects of cultivars origin change on physical and biochemical quality in two pomegranate cultivars (mean of the 2-year data).

The findings reveal that the fruit weight values vary from 252 to 215 g, and the aril fruit weight values range from 116 to 147 g (Fig. 2). This range is consistent with previous reports from other collections cultivated in various areas across the globe. The fruit weight level in various Italian pomegranate accessions varied from 107 to 238 g/100 g^11^, but the fruit weight level in pomegranates gathered in other countries ranged from 162 to 339 g. The fruit weight data revealed considerable variances between cultivars grown in various climates. Cultivar responses to changing origin differed, such that the largest fruit weight in MMS was connected to the origin climate, but RPGN were not statistically different in both climates (Fig. 2).

The impact of changing origin on skin weight was cultivar-dependent. For example, the most significant skin weight in RPGN cultivars indicated a warm temperature, but the highest skin weight in the MMS cultivar indicated an origin climate (Fig. 2). Also, the maximum aril weight was related to MMS in the origin climate and RPGN in the origin climate, whereas the minimum aril weight was shown for RPGN and MMS in a warm climate (Fig. 2). The most fabulous edible part 58.55% was associated with MMS in the origin climate, RPGN in the origin climate, RPGN in the warm climate, and MMS in the warm climate, respectively (Fig. 2).

### Biochemical properties

In the origin climate, both pomegranate cultivars, RPGN and MMS had the most significant antioxidant levels (52.61and 38.4%, respectively) (Fig. 2). However, each cultivar’s lowest antioxidant value (31.46 and 46.76%) was found in Yazd's warm environment (Fig. 2).

Furthermore, this research found that pomegranate fruits from the original environment (mountain climate) had the highest total phenol content in both cultivars, while those from milder climates had the lowest (Fig. 2). The biochemical experiment findings revealed that the vitamin C content of pomegranates varied greatly depending on the temperature. A considerable variance in vitamin C levels between growing areas in each cultivar might be attributed to climatic changes between the growing regions. Furthermore, our findings revealed that fruits from warm climates and origin locations had the highest levels of vitamin C in both cultivars. In addition, pomegranates from 'MMS' had higher vitamin C levels than RPGN cultivars from the same region and environment (Fig. 2).

Both cultivars’ anthocyanin levels plummeted in a warm environment. RPGN (14.64 mg 100 g^–1^) and MMS (25 mg100 g^–1^) had the greatest anthocyanin content among the fruits grown in the origin environment, while the lowest anthocyanin content was found in pomegranate cultivars from warm climates (4.04 and 9.90 mg 100 g^–1^) (Fig. 2).

### Juice quality

**Figure 3:**
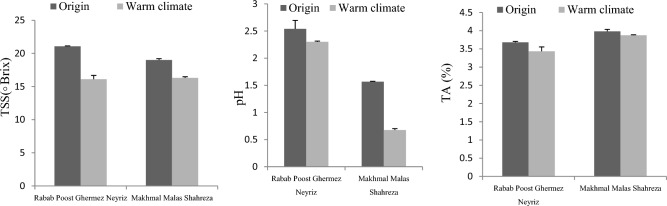
Effects of cultivars origin change on juice quality in two pomegranate cultivars (mean of the 2-year data).

Our study shows that pomegranate cultivar origin had an apparent influence on fruit juice quality because pomegranates originating from two different climate regions showed statistically different TSS, TA, and pH values; and the high TSS and pH values of pomegranates produced in the origin climate than in the warm climate (Fig. 3). The change in growing location had a substantial impact on the TA juice. The maximum pH and TA were detected in both cultivars cultivated in the origin environment, whereas the lowest pH and TA were observed in the warm climate. TA ranged from 3.68 to 3.98% in origin locations for RPGN and MMS cultivars, respectively. However, in warmer conditions, TA levels plummeted to 0.25 and 0.11%, respectively. TSS juice of pomegranate differed significantly between the two cultivars grown in different climates (Fig. 3). Both cultivars plummeted TSS levels in a warm environment, whereas RPGN had the greatest decreasing rate of TSS (21.06 to 19◦Brix) in warm climates than the origin location.

### Correlation analysis

**Figure 4:**
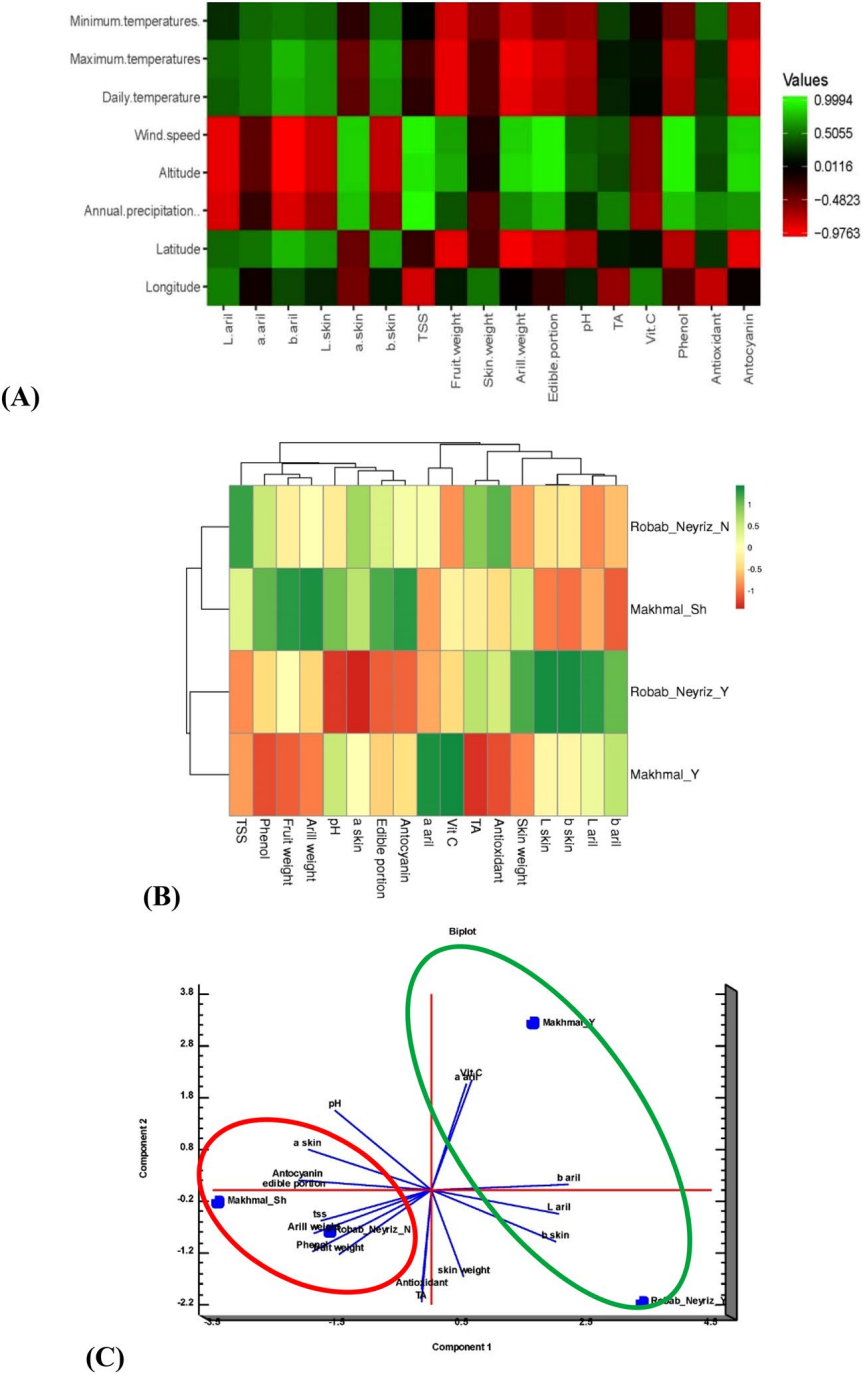
The correlation heatmap of the studied traits and the geographical characteristics (**A**), the behavior of the studied traits in different environments and different cultivars (**B**) and the principal component analysis (PCA) of the studied traits in different treatments (**C**) (mean of the 2-year data).

Based on this data, minimum temperatures had a significant positive correlation with a*aril, b* aril, L* skin, b* skin, TA, and antioxidants. Furthermore, it has also had the highest negative correlation with fruit weight, aril weight, edible portion, pH, phenol, and anthocyanin content. Other climate factors such as maximum temperatures and the daily temperature had the highest significant positive correlation with antioxidants, and all color coordinates in skin and aril except a* skin. Also, some geographical factors, such as wind speed, altitude, and annual precipitation, had a significant positive correlation with a* skin, TSS, fruit weight, aril weight, edible portion, pH, TA, phenolic compound, antioxidants, and anthocyanin content, whereas these geographical factors had a significant negative correlation with the majority of index color in skin and aril (L* aril, b* aril, L* skin, b* skin) and vitamin C. In addition, all index colors in arils (L*, a*, b* aril), L* skin, b* skin, and antioxidant capacity have positive correlations with latitude, whereas latitude has a negative and significant correlation with fruit weight, aril weight, edible part, pH, phenolic compounds, and anthocyanin concentration. Longitude was shown positively affected on L* aril, b* aril, L* skin, skin weight, and vitamin C; however, a highest negative relationship was found between TSS, antioxidant capacity, TA, and a* skin, respectively (Fig. 4).

### The cluster analysis

According to the heatmap (Fig. 4), two groups have been formed in various places based on the investigated traits. As a result, the primary regions were positioned near each other, as were the secondary regions (cold climate). RPGN and MMS cultivated in origin location were grouped in the same group in the first group (Fig. 4). RPGN and MMS cultivars grown in desert climate were also grouped in the same group in the second group. RPGN planted in origin was superior to the same cultivar cultivated in warm climate in terms of most morphological traits, and except for five traits, b aril, L aril, b skin, L skin, and skin weight, had absolute superiority in most traits, according to the heat map and the studied traits. Compared to the same cultivar in Yazd, MMS grown in Shahreza had a similar scenario. In just two traits, vitamin C and an aril were the Makhmal cultivar grown in Yazd superior to the same cultivar cultivated in Shahreza, and in all other traits, the total supremacy belonged to the MMS produced in Shahreza. This demonstrates the advantages of the original areas over desert or warm locations for crop growth and development (Fig. 4).

### Principal component analysis

It was also used to assess the behavior of the traits in various locations. The results of principal component analysis corroborated the findings of cluster analysis perfectly (Fig. 4). As a result, the areas of origin were put near one another, as were the warm climatic zones. RPGN, grown in Neyriz, and MMS, farmed in Shahreza, was grouped in the first group. Furthermore, PCA findings revealed that, except for b* aril, L* aril, b* skin, L* skin, skin weight, and vitamin C, both cultivars farmed at their birthplace had total superiority in most characteristics. This demonstrates the advantage of the origin areas over the warm environment for crop growth and development. Furthermore, the shift in the origin site of pomegranate cultivars substantially impacted juice quality since both cultivars cultivated in the original location had higher TSS, TA, and pH values than warm climate cultivars. Some climatic parameters, such as maximum and daily temperatures, exhibited the most significant positive link with antioxidants and all color coordinates in skin and aril except a* skin. Furthermore, some geographical factors, such as wind speed, altitude, and annual precipitation, had a significant positive correlation with a* skin, TSS, fruit weight, aril weight, edible portion, pH, TA, phenol compounds, antioxidants, and anthocyanin content. In contrast, these geographical factors had a significant negative correlation with the majority of the index colors in skin and aril (L* aril, b* aril, L* skin, b* skin) and vitamin C.

## Discussion

Color is one of the essential characteristics of pomegranates, and it is crucial for customer selection and marketability of pomegranate fruit xenia^12^. The fruit of RPGN and MMS cultivated in the native climate, with a more vivid red color and a homogenous cover of skin fruit color, attracts greater commercial attention. However, fruits cultivated in Yazd province (warm environment) had unsuitable skin and aril color and were weaker in red coordinate (a*). Our findings agreed with recent research that climatic conditions influence the color of pomegranate fruits produced in a desert setting^13^.

The deep red color of the aril and skin fruit of both pomegranate cultivars from the origin climate was most likely due to lower temperatures, especially during September and October, compared to those from the Yazd region which has a warm and desert environment. Furthermore, it was considered that the reduced vivid red color in aril and a homogenous cover in skin fruit at raised temperatures in the warm environment compared to the origin climate were attributable to accelerated pigment degradation^14^. These data revealed that the reaction of cultivars to change origin differed, with MMS being less susceptible to change in origin and climatic change of fruit and aril color than RPGN. The findings indicate that the genetic background of these cultivars had a significant role in fruit color.

Our findings revealed that changing the original climate of pomegranate cultivars affects fruit weight, aril weight, and skin weight. Similar findings were reported in earlier studies revealing that climatic circumstances impact the arils and fruit weight of pomegranate fruits cultivated in various climates^13,15^. In contrast, Boussaa et al.^16^ discovered that the growing site did not affect fruit weight in Turkish pomegranate cultivars. The findings revealed that pomegranate fruits cultivated in the mountains had more incredible fruit and aril weights than those grown in desert climates. According to Schwartz et al.^15^, pomegranate fruits cultivated in a desert climate generate fewer arils than those grown in Mediterranean climate settings. The findings of the edible part measures were consistent with the aril and skin weight (Fig. 2), and the shift in temperature from the original climate to the warm environment influenced the edible portion. According to Pagliarini^17^, the edible part might be a valuable element in economically classifying pomegranate fruit for the processing sector, since for the same weight of fruit, the customer selects the fruit pomegranate with the most edible portion. These findings imply that fruits cultivated in climates other than the origin environment (hot, dry climate) have a decreased edible fraction in pomegranate fruits. Prior research evaluating Tunisia cultivars in various regions found that pomegranate fruits from the center of Tunisia’s climate had the most extraordinary aril production (62.74 percent), while the lowest (45.02 percent) was associated with the southern Tunisian climate^16^. Previous research found that the edible portion of pomegranates was correlated with the length and width of the arils^18^. We now propose that the environmental conditions in Yazd, precisely the temperature and humidity, promote a decrease in aril growth, which is the main reason for the low edible portion found in fruits from this habitat.

According to this research, temperature shift from the origin climate to another place dramatically reduces the antioxidant capacity of pomegranates. Reported similar findings, Schwartz, et al.^15^demonstrated that environmental circumstances significantly impact pomegranate antioxidant activity. In contrast, previous research in Tunisia found no change in DPPH activity in pomegranates from various regions. Consequently, pomegranate fruits cultivated in mountain climates are of more outstanding quality than those grown in desert climates. Previous research has shown that pomegranate fruits cultivated in arid climates had lower antioxidant content in aril juice than those grown in Mediterranean climates^15^. Furthermore, under pomegranate growing settings, a temperature of 25–30 °C during fruit development might considerably increase antioxidant activity in pomegranates^19^. A substantial variation in total phenol content was also identified across growing sites; comparable findings demonstrated that the climatic conditions in growing locations significantly influenced total phenol content in pomegranate fruits^16^. Consistent with our earlier findings, Schwartz et al.^15^ found that total phenol in aril juice was lower in desert-grown fruit than in Mediterranean, subtropical fruit. However, Attanayake, et al.^20^ discovered that the total phenol concentration of pomegranate arils was much lower in cold and rainy areas of Sri Lanka. Large environmental component changes throughout their investigation most likely led to such changes. We also believe that total phenol content, which is affected by environmental circumstances such as light, radiation, temperature, and genetic background, is the most significant factor in phenol production. In this study vitamin C concentration varied from 4.93 to 54.49 mg/ml^–1^ (Fig. 2), higher than Mditshwa et al.^12^. Furthermore, our findings revealed that fruits from warm climates and origin locations had the highest levels of vitamin C in both cultivars. Previous research revealed that the vitamin C content of pomegranate aril is regulated by the climate and growing site^12^. This contradicted previous findings that the ascorbic acid concentration in pomegranate cultivars did not change considerably across climates^15^. Other research indicates that genetic variation and the climatic area cultivated influence the vitamin C value of pomegranate fruit^21,22^.

There were substantial changes in aril color between pomegranate fruits from the origin region and the warm environment, as shown by the anthocyanin value measurements (Fig. 2), which were consistent with the color observations. According to Mditshwa et al.^12^, pomegranate fruit color development is influenced by anthocyanin concentration. Thus, low anthocyanin content in the aril and skin fruit of desert climate may be linked to the poor red color, whereas the greatest L* index (red-colored) in the skin and aril of Neyriz and Shahreza climatic origin fruits may be linked to an increase in anthocyanin content^23,27^. Pomegranate cultivars cultivated in Mediterranean climates had greater anthocyanin concentrations in fruit arils than those produced in desert climates, according to Schwartz et al.^15^. However, earlier research on gene expression analyses anticipated that the overall anthocyanin content in the Teldeniya area would be lower than samples obtained from other places because of the drier and warmer temperature^20^. Red-skinned apples with higher anthocyanin content had lower anthocyanin biosynthetic gene expression when the temperature was increased, according to Wang et al.^24^. Anthocyanin production genes in strawberries (FaDFR, FaANS, FaUFGT, and FaMYB10) were significantly reduced under a high air temperature regime of 30/15 °C, day/night in comparison to a control (20/15 °C, day/night)^25^. Recent research indicated that climatic circumstances had a significant influence on the pH and TA levels of several pomegranate accessions, with TA levels dropping to 0.13%in a warm environment^26,27^. These results were comparable to those published previously for pomegranate, indicating that increasing the temperature lowered TA in aril juice owing to a decline in malic acid levels at warm temperatures^28^. Similarly, TA fruit juice dropped when the temperature rose due to deceased malic acid concentration in peach fruit^29^ and grape berries^30^. Thus, our findings revealed that the genetic background of these cultivars had a significant role in TSS. Similar findings were reported in earlier studies revealing that climatic circumstances impact the arils and fruit weight of pomegranate fruits cultivated in various climates^12,15,31^. Our findings agreed with Schwartz et al.^15^, pomegranate fruits cultivated in a desert climate produce fewer TSS than those grown in another climate.

It has also had a significant negative relationship with fruit weight, aril weight, edible portion, pH, anthocyanin content, and phenolic compounds (Fig. 4). A previous pomegranate study revealed a substantial negative association between temperature and total phenolic content^16^. Furthermore, there was a link between mean annual temperature and metabolic fruit and juice properties such as total phenolic content and antioxidant activity, as well as in pomegranate peel and arils^20^. Fruit cultivars from mountain locations, for example, had higher fruit weight, aril weight, edible portion, pH, TA, TSS, total phenol compound, antioxidant capacity, and anthocyanin content than those from desert regions. Our findings are consistent with prior research, which revealed that hot Mediterranean regions yield higher-quality pomegranates than desert ones^3^. Other research, however, found that the total phenol compound in the fruit peels of most kinds gathered in dry regions was greater than in those collected in Mediterranean climates^32^. Several studies have shown a considerable association between local climatic region and physicochemical features of fruits and vegetables. Similar results were reported by Mditshwa et al.^12^, who discovered that total phenol and antioxidant activity were linked to rainfall. Boussaa et al.^16^ discovered a positive relationship between antioxidant activity and rainfall. Our results support previous studies that linked total phenol, total antioxidant, TSS, and vitamin C content to microclimate rainfall^33,34^. These results showed that pomegranate fruit grown at 1845 m (Shahreza) and 1632 m (Neyriz) altitude contained higher antioxidants capacity; total phenol compounds, and red color in aril than those growing at 135 m (Yazd as warm climate).These findings were similar to those of Mditshwa et al.^12^ they reported that pomegranate fruit from lower latitudes had higher total phenolic content than fruit from high altitudes. According to Mditshwa et al.^12^, altitude has a significant impact on the phenolic biosynthesis pathway in pomegranate fruits. Furthermore, according to the heatmap analysis, sort of cluster might contribute to a better understanding of which climatic conditions and geographical variables have the most significant influence on pomegranate fruit quality. This analysis demonstrates the advantages of the original areas over desert or warm locations for crop growth and development.

In addition, both cultivars grown in Yazd were clustered in the second group. It was previously shown that the principal component analysis might demonstrate how environmental circumstances impact biochemical changes in distinct locations in pomegranate fruit^15,16^. Furthermore, PCA analysis indicates that climatic conditions and geographical variables influence physical, biochemical, and quality changes in various locations. This demonstrates the advantage of the origin areas over the warm environment for crop growth and development. Furthermore, the shift in the origin site of pomegranate cultivars substantially impacted juice quality since both cultivars cultivated in the original location had higher TSS, TA, and pH values than warm climate cultivars.

## Conclusion

Our findings revealed that changing the origin climate of pomegranate cultivars influenced fruit color, fruit weight, aril weight, skin weight, and edible parts and those cultivar responses to changing origin differed. This research found that pomegranate fruits from the original environment (mountain climate) had the highest biochemical attributes than pomegranate fruits from a warm climate. Furthermore, the shift in the origin site of pomegranate cultivars substantially impacted juice quality since both cultivars cultivated in the original location had higher quality than warm climate. Some climatic parameters, such as maximum and daily temperatures, exhibited the most significant positive link with antioxidants and all color coordinates in skin and aril except a* skin. Furthermore, some geographical factors, such as wind speed, altitude, and annual precipitation, had a significant positive correlation with a* skin, TSS, fruit weight, aril weight, edible portion, pH, TA, phenol, antioxidants, and anthocyanin content. In general, we propose that climatic conditions and geographical characteristics be addressed when selecting cultivars and developing pomegranate orchards in various climates, especially in warmer climes than the cultivars' origin environment.

## Materials and method

### Plant material

This experiment was performed during two growing seasons (2019–2020 and 2020–2021) on two pomegranate cultivars, RPGN and MMS were used in this experiment. The RPGN is an Iranian commercial cultivar with ample fruit, crimson skin and aril, and a sweet–sour flavor. It is also a Neyriz area native. MMS is an Iranian cultivar native to Shahreza in Esfahan province, with ample fruit, red skin and arils, and a sweet–sour flavor. A broad range of ripening dates, peel, and aril colors and tastes were considered while selecting these cultivars. Each cultivar in the native region (Neyriz region for RPGN and Shahreza region for MMS) and other warm regions in the pomegranate collection of the Yazd agricultural and natural resources research center, Yazd province in central Iran with a desert climate was evaluated in this study. The Neyriz experimental orchard is situated on the eastern side of Bakhtegan Lake, some 220 km southeast of Shiraz. Neyriz is situated in the Zagros mountain ranges of southern Iran and has a moderate mountainous climate (Table 2). The Shahreza experimental orchard is approximately 80 km south of Isfahan, and the Zard Kooh mountain range spans from the north to the southeast of the city. Shahreza is situated in central Iran and is part of the Zagros mountain ranges; hence it has a chilly mountainous climate. Trees in the experimental orchards were planted at a 4 × 5 m distance with three replications per location. The experiment and horticultural management were carried out following the recommended orchard practices by the Iranian Ministry of Jihad-e-Agriculture. Ten fruits from each experimental group were harvested when commercially mature by an experienced horticulturist and transferred to the laboratory.

**Table 2:** Geographic/environmental data of three studied regions (mean of the 20-year data).

Location	Province	Minimum temperature (°C)	Maximum temperature (°C)	Mean daily temperature (°C)	Longitude (°)	Wind speed (knot)	Annual precipitation (mm)	Altitude (m)
Yazd	Yazd	11.7	26.5	19.2	54.35	5.1	59.2	135.0
Shahreza	Isfahan	7.0	22.3	14.7	51.58	5.3	142.2	1845.0
Neyriz	Fars	13.4	26.4	19.4	54.32	5.3	204.9	1632.0

### Meteorological parameters and soil parameters of the locations

Table 1 presents each experimental orchard’s original geographic and climatic data (Spanning 20 years), including altitude, minimum and maximum temperatures, mean daily temperature, average wind speed, and average annual rainfall from standard meteorological stations in Iran experimental orchards were collected. No rainfall was reported during the growth seasons at any of the three study sites.

### Measurement of fruit physical properties

A digital scale with an accuracy of 0.001 g was used to measure the fruit’s fresh weight. After measuring the fruit’s fresh weight, the husks were carefully chopped in the equatorial zone with a sharp knife. The peel and arils were physically removed from the fruit to determine the skin weight, aril weight, and edible part. The edible part of the fruit was calculated using the method below^2^:

$$\mathrm{Edible\,\, portion\,\, of\,\, fruit }\left(\mathrm{\%}\right)=\frac{\mathrm{fruit \,\,weight}-\mathrm{ skin\,\, weight }}{\mathrm{fruit \,\,weight}}\times 100.$$

Fruit and aril color (as L*, a*, and b*) was measured using a chromameter (Chroma Meter CR-400, Konica Minolta, Japan). The color parameters represent whiteness or brightness/darkness (L*), redness/greenness (a*), and yellowness/blueness (b*).

### Measurement of biochemical properties

#### Total soluble solids, pH, and titratable acidity (TA)

Fresh pomegranate arils were utilized to evaluate biochemical parameters such as pH, TSS, and TA, as well as phenol, antioxidants, and vitamin C. Total soluble solids (°Brix) in the juice, were calculated by extracting and mixing one drop of juice from each fruit into a digital refractometer (Atago NI, Japan) at 20 °C and displaying the results as a percentage. TA was represented as citric acid content (g.100ml^–1^) and was determined by titrating juice samples (5 mL) with 0.1 N NaOH to the titration endpoint of pH 8.2, which was monitored using a pH meter (Labtron). The pH of the aril juice samples was homogenized and measured at room temperature using a pH meter (WTW 526, Germany), which had previously been calibrated to a pH range of 4 to 7.

#### Ascorbic acid

A spectrophotometer was used to quantify ascorbic acid against a standard curve that included 0.0025 percent of 2, 6-dichlorophenolindophenol (DCPIP) dye and 1 percent of metaphosphoric acid^35^.

#### Total phenol content

The total phenol concentration in juice samples was evaluated using the Folin-Ciocalteu (Folin-C) and colorimetric methods established by Singleton and Rossi^36^. By applying the Folin-Ciocalteu reagent to the juice sample, the level of total phenol was measured using a spectrophotometer at 750 nm. The mean value (mg) of gallic acid equivalents (GAEs) per mL of crude juice was used to calculate the results.

#### Antioxidant activity

Antioxidant activity was assessed using a commercially available free radical DPPH (2, 2-diphenyl-1-picrylhydrazyl)^37^. In brief, 0.1 mL of pomegranate juice was combined with 0.9 mL of 100 mM Tris–HCl buffer (pH = 7.4), followed by 1 mL of DPPH (500 M in ethanol). The control sample was made the same way but with 0.1 ml of distilled water instead of pomegranate juice. The mixtures were violently mixed and allowed to stand for 30 min. A spectrophotometer was used to measure the absorbance of the resultant solution at 517 nm. For background correction, the reaction mixture without DPPH was employed.

#### Total anthocyanin

Total anthocyanin was determined spectrophotometrically as described by^38^. An aliquot of juice (2 mL) was diluted with a pH 1 solution to a volume of 25 mL (125 mL of 0.2 M KCl and 375 mL of 0.2 M HCl). A second portion (2 mL) was diluted to 25 mL using buffered solutions with a pH of 4.5 (400 mL of 1 M sodium acetate, 240 mL of 1 M HCl, and 360 mL of H_2_O). The solutions’ absorbance was measured at 510 nm.

### Statistical analysis

All data were presented as an average of two years of study. An analysis of variance (ANOVA) was performed using SAS version 9. Differences in means were tested for significance using Duncan’s multiple range tests at p < 0.05. The findings were presented as means ± standard error.

### Ethical approval

We confirm that all the experimental research and field studies on plants (either cultivated or wild), including the collection of plant material, complied with relevant institutional, national, and international guidelines and legislation. All of the material is owned by the authors and/or no permissions are required.

## Data Availability

Some or all data, models, or code that support the findings of this study are available from the corresponding author upon reasonable request.
